# Multidisciplinary Content Validation of the GiriMok Point-of-Care Protocol for Neonatal Cyanotic Congenital Heart Disease

**DOI:** 10.7759/cureus.109877

**Published:** 2026-05-29

**Authors:** Girish Gupta, Syed Moiz Ahmed, Richa Joshi, Shantanu Shubham

**Affiliations:** 1 Neonatology, Graphic Era Institute of Medical Sciences, Dehradun, IND; 2 Neonatology and Pediatrics, Graphic Era Institute of Medical Sciences, Dehradun, IND

**Keywords:** clinical decision support, content validity, content validity index, echocardiography, iterative validation, neonatal cyanotic congenital heart disease, point-of-care protocol, prostaglandin e₁, pulse oximetry

## Abstract

Background

Neonatal cyanotic congenital heart disease is a time-critical emergency requiring immediate bedside decision-making. Current guidelines lack a validated, comprehensive point-of-care (POC) protocol suitable for frontline clinicians in diverse settings. The GiriMok Protocol was developed to bridge this gap. This study aimed to develop and establish the content validity of the GiriMok POC Protocol through a rigorous four-phase iterative multidisciplinary expert validation process.

Methodology

This four-phase study comprised two external validation phases and two author-led refinement phases. Phase 1 involved 18 multidisciplinary experts using a 33-parameter mixed-methods instrument. Phase 2 consisted of focused revalidation by five specialists (neonatologists and a pediatric cardiologist) using a 16-item instrument with modified kappa (k*) computation. Phases 3 and 4 involved evidence-referenced enhancements against 2023-2025 guidelines and final quality assurance. Content Validity Index (CVI) was calculated at item (I-CVI) and scale (S-CVI/Ave) levels.

Results

In total, 18 unique validators participated across 24 episodes. Phase 1 yielded an S-CVI/Ave of 0.927, with all items meeting the I-CVI ≥0.78 threshold and zero safety concerns. Phase 2 achieved perfect scores (S-CVI/Ave = k* = 1.000) following the integration of a 22-item revision agenda. In total, 35 expert-informed corrections resulted in the final publication-ready GiriMok V4.0.

Conclusions

The GiriMok POC Protocol achieved unanimous multidisciplinary validation. The perfect Phase 2 CVI demonstrates methodological rigor and responsiveness to expert feedback. The protocol is now positioned for prospective clinical evaluation and national dissemination as a neonatal emergency decision-support tool.

## Introduction

Clinical emergency context

Cyanotic congenital heart disease (CHD) represents one of the most time-critical diagnostic and management challenges in neonatal medicine. Globally, CHD affects approximately eight per 1,000 live births, with cyanotic lesions comprising roughly 20-25% of all cases, accounting for a significant proportion of neonatal morbidity and cardiac-related mortality in the first week of life [1,2]. Despite advances in pediatric cardiac surgery and critical care, outcomes remain heavily dependent on the timeliness and accuracy of initial point-of-care (POC) recognition and stabilization, particularly in resource-limited and non-tertiary settings [3].

The clinical presentation of cyanotic CHD in the neonate is often non-specific and overlaps with non- cardiac causes of hypoxemia, including respiratory distress syndrome, persistent pulmonary hypertension of the newborn (PPHN), and sepsis. This diagnostic ambiguity, combined with the absence of structured, bedside-friendly algorithms, creates a critical gap between the onset of hemodynamic compromise and definitive intervention. Pulse oximetry-based newborn screening has improved early detection [4-6], yet a validated, practitioner-oriented protocol that integrates clinical assessment, bedside investigations, and initial pharmacological stabilization, notably prostaglandin E₁ (PGE₁) [7,8] in a systematic POC framework, remains unavailable for widespread adoption in Indian and similar low and middle-income country (LMIC) settings [9].

Rationale for a structured point-of-care protocol

In India, the burden of undetected or inadequately stabilized cyanotic CHD translates into preventable mortality and long-term morbidity, disproportionately affecting non-tertiary facilities [9]. Existing international guidelines, including neonatal resuscitation frameworks [10] and CHD management guidelines applicable to tertiary and subspecialty practice [11,12], are comprehensive for specialist centers but do not address the bedside, real-time, sequential decision needs of frontline clinicians in primary and secondary care settings, particularly in resource-limited contexts. Structurally, no single validated instrument cohesively integrates all five clinical functions essential at the point of first contact: bedside screening (pulse oximetry algorithm), differential diagnosis (cyanotic versus non-cyanotic, cardiac versus pulmonary), emergency pharmacological management (PGE₁, iNO, supportive care), diagnostic interpretation (chest X-ray, ECG, echocardiography), and transfer decision-making. This five-function integration gap for the undifferentiated cyanotic neonate is precisely what the GiriMok Protocol is designed to fill.

Content validity as the appropriate methodological framework

Content validity, the degree to which a tool comprehensively and accurately represents the clinical construct it claims to address, is the foundational validity requirement for clinical instruments, preceding criterion and construct validity assessment [13,14]. The Content Validity Index (CVI), operationalized through item-level CVI (I-CVI) and scale-level CVI (S-CVI/Ave), is the most widely recommended quantitative methodology for establishing content validity in health sciences [15,16]. An iterative, multi-phase validation design incorporating diverse expert perspectives and systematic protocol refinement in response to feedback represents the methodological standard for complex clinical instruments [17,18]. The present study was designed to meet this standard.

The four-phase iterative design

The GiriMok Protocol was developed and validated through four sequential phases: a broad multidisciplinary expert consultation (Phase 1), a specialist CVI panel (Phase 2), an author-led protocol enhancement (Phase 3), and a precision quality-assurance closure (Phase 4). This progressive refinement architecture allowed iterative incorporation of expert insights, ensured that the final protocol reflected both quantitative consensus and qualitative clinical wisdom, and produced a tool of demonstrably high content validity.

Study objectives

The primary objective was to establish the content validity of the GiriMok POC Protocol through a four-phase iterative expert panel study, with the specific aim of achieving pre-specified CVI thresholds (S- CVI/Ave ≥ 0.90; I-CVI ≥ 0.78) as recommended by Polit and Beck [14,15]. Secondary objectives were to document expert-recommended refinements, to quantify iterative improvement across phases, and to produce a protocol version suitable for immediate clinical and educational implementation.

The GiriMok Protocol: architecture, content development history, and nomenclature

The GiriMok protocol was conceptualized and developed by the principal innovator at Graphic Era Institute of Medical Sciences (GEIMS), Dehradun. The name “GiriMok” derives from the inventor’s surname (Giri) and the clinical context (Mok: Management of cyanosis/cardiac disease). The protocol underwent sequential versioning from V1.0 through V4.0, with each version incorporating evidence updates and expert-recommended refinements.

Protocol architecture

GiriMok V4.0 is organized into 11 clinical sections, an abbreviations glossary, and a clinical pearls and pitfalls compendium. The architecture follows a sequential management logic, from newborn screening through immediate stabilization, bedside diagnosis, targeted investigations, emergency pharmacotherapy, arrhythmia management, PPHN differentiation, POC echocardiography, and transfer preparation.

Key innovations

GiriMok incorporates several structural innovations absent from existing bedside tools. The five C’s differential diagnosis framework provides a structured five-category differential that systematically excludes life-threatening non-cardiac mimics before committing to cardiac diagnosis. The PGE₁ contraindication clarification establishes, based on pathophysiological review, a single absolute contraindication (obstructed total anomalous pulmonary venous connection (TAPVC)), replacing the clinically confusing multiple contraindication lists in many textbooks. The PPHN differentiation algorithm enables simultaneous evaluation for the two most important causes of neonatal hypoxaemia. POC echocardiography integration provides a structured bedside echo protocol accessible to trained neonatologists without requiring formal cardiology input for initial assessment.

## Materials and methods

Study design and setting

A four-phase iterative content validation study was conducted at the Department of Neonatology, GEIMS, Dehradun, India. The study constitutes a tool development and content validation exercise classified as exempt from full institutional ethics committee review, consistent with applicable institutional guidelines governing quality improvement and educational tool development activities. No patient data were collected at any stage.

The protocol’s clinical sections, abbreviations glossary, and a clinical pearls and pitfalls compendium are presented in Table 1.

**Table 1:** Architecture of the GiriMok POC Protocol V4.0. BSA = body surface area; CCHD = critical congenital heart disease; PGE₁ = prostaglandin E₁; TAPVC = total anomalous pulmonary venous connection (standardized throughout; TAPVR = total anomalous pulmonary venous return is an older synonym, now deprecated in favour of TAPVC); PPHN = persistent pulmonary hypertension of the newborn; POC = point-of-care; SpO₂ = oxygen saturation; SVT = supraventricular tachycardia; TR = tricuspid regurgitation

Section	Title	Key clinical content
Section 0	Pulse Oximetry Screening	CCHD screening algorithm; pre/post-ductal SpO₂ protocol; cut-off values; repeat testing; 2025 AAP criteria; referral thresholds
Section 1	Immediate Stabilization	ABCDE approach; thermoregulation; IV access; monitoring parameters; emergency escalation
Section 2	Hyperoxia Test	Step-by-step protocol; PaO₂ interpretation thresholds; grey-zone management; limitations; role vis-à-vis pre/post-ductal SpO₂
Section 3	Clinical Assessment Framework	Physical examination; cardiac vs. pulmonary differentiation signs; differential cyanosis; dysmorphic feature screen
Section 4	Differential Diagnosis: Five C’s Framework	Five-category differential: CCHD; cardio-circulatory shock; chest/respiratory; cerebral; cellular (methaemoglobinemia); lesion-specific SpO₂ targets
Section 5	Investigations	Tiered investigation ladder: mandatory vs. conditional; ECG interpretation; CXR signs (including Snowman sign — unobstructed TAPVC ); ABG; echocardiography prioritisation
Section 6	Management: PGE₁ Protocol	Dosing algorithm; titration; single absolute contraindication (obstructed TAPVC); relative precautions; side-effect monitoring; adjunct therapies
Section 7	Arrhythmia Recognition & Management	Neonatal ECG rhythm recognition; SVT, heart block, QTc prolongation; cardioversion guidance
Section 8	PPHN vs. CCHD Differentiation	Structured differentiation algorithm; echocardiographic differentiators; hyperoxia integration; management divergence decision points
Section 9	POC Echocardiography	Structured POC echo views; BSA-indexed Ebstein’s anomaly criteria; great vessel nomenclature; TR jet units; segmental analysis; reporting template
Section 10	Transfer Preparation	Pre-transfer stabilization checklist; communication protocol; documentation; transport equipment; PGE₁ infusion continuation
	Abbreviations Glossary	70+ abbreviations defined; clinical and pharmacological terminology
	Clinical Pearls & Pitfalls	Evidence-based pearls; common diagnostic errors; high-risk scenarios; memory aids

Protocol development and versioning

The index tool GiriKous V1.0, redesignated GiriMok V2.0 following Phase 1 revision to reflect expanded clinical scope, was developed by the principal innovator (GG) as a comprehensive POC clinical reference for bedside management of a cyanotic neonate. Version designations: V1.0 (Phase 1 entry), V2.0 (Phase 2 entry), V3.0 (Phase 3 entry), V4.0 (final, post-Phase 4 quality assurance).

Four-phase validation architecture

The complete study architecture is summarized in Table 2. Phases 1 and 2 constituted external expert validation. Phases 3 and 4 constituted author-led refinement and quality assurance, respectively.

**Table 2:** Summary of four-phase validation architecture. Phases 1–2 constitute external validation phases. Phases 3–4 constitute author-led refinement phases. Role codes (N1, PC1, N2, N3, E1) used in tables denote the sequential order of participation, not specialty classification. Full names are listed in Acknowledgements. CVI = Content Validity Index

Phase	Activity	Validators (n)	Version	Instrument	Primary outcome
1	Broad multidisciplinary validation: GiriKous V1.0	18 (external)	V1.0 → V2.0	33-parameter structured instrument (16-item Likert CVI + 6 evidence checklist + 5 recommendations + 6 qualitative domains)	S-CVI/Ave = 0.927; 22 revision items identified
2	Focused specialist revalidation: GiriMok V2.0	5 (external)	V2.0 → V3.0	16-item CVI instrument; 4-point Likert scale; modified kappa (k*)	S-CVI/Ave = k* = 1.000; unanimous consensus; 0 new corrections
3	Enhancement integration: GiriMok V3.0	0 (author-led)	V3.0	Evidence-referenced revision log; international guidelines 2023–2025	Eight enhancements integrated
4	Principal innovator QA: GiriMok V4.0 FINAL	1 (author QA)	V4.0 FINAL	Expert clinical precision review	Five precision corrections applied; V4.0 declared final
Total	GiriMok V4.0 FINAL	18 unique external validators; 23 validator-participation events; 24 validation episodes total (5 Phase two validators drawn from Phase one panel)	V1.0 → V4.0	—	35 expert-informed corrections; GiriMok V4.0 publication-ready

Phase 1: Broad multidisciplinary validation (validator recruitment and panel composition)

A purposive multidisciplinary expert panel of 18 clinicians with direct relevance to the clinical domain was assembled. The panel included nine neonatologists (50.0%), six pediatricians (33.3%), two pediatric cardiologists (11.1%), and one adult cardiologist (5.6%), with a mean clinical experience of 3.6 years at the specialist level (SD = 0.51; range = 3-4 years after the highest qualification). While the panel predominantly comprised early-career specialists, this was considered methodologically relevant because the protocol is specifically intended for frontline clinicians who frequently manage cyanotic neonates in first-contact and resource-limited settings. All 18 validators declared no conflict of interest (100%). Validators were provided the complete GiriKous V1.0 protocol and the validation instrument, with a structured covering note outlining the purpose, timeline, and instructions. One validator (Validator 1) completed the quantitative Likert matrix only; 17 validators completed all instrument components, including categorical and qualitative sections.

Validation Instrument

Phase 1 employed a 33-parameter structured mixed-methods instrument: a 16-item four-point Likert CVI matrix spanning five domains (content validity, visual design, clinical usability, educational value, overall quality); a six-item evidence-based checklist; four categorical recommendation items (Yes/No/Yes with modification); and six open-ended qualitative feedback domains.

Quantitative Analysis

I-CVI was calculated for each of the 16 Likert items as the proportion of validators rating the item ≥3 (Agree or Strongly Agree), following the method of Lynn [13] and Polit and Beck [14]. S-CVI/Ave was computed as the arithmetic mean of all 16 I-CVI values. Acceptance thresholds were pre-specified as I-CVI ≥0.78 per item and S-CVI/Ave ≥0.90 overall. Items with I- CVI ≥0.78 but <0.90 were flagged for targeted revision.

Qualitative Analysis

Open-ended validator responses were analyzed using an inductive descriptive thematic approach focused on actionable protocol refinement. Narrative feedback was independently reviewed by the principal innovator and categorized according to the primary nature of the concern raised. Comments were coded into the following four operational thematic domains: (1) factual inaccuracies or outdated evidence, (2) essential missing clinical content, (3) structural or usability barriers affecting bedside implementation, and (4) supplementary enhancement recommendations. Recurrent suggestions from multiple validators were consolidated into unified revision items to prevent duplication. Each coded item was prospectively entered into a traceable revision log and mapped directly to corresponding protocol modifications incorporated into subsequent protocol versions. Thematic categories were mapped to a structured revision agenda that informed V2.0 development.

Phase 2: Focused specialist revalidation

Phase 2 was conducted during January-February 2026, following a comprehensive revision of the protocol to produce GiriMok V2.0, incorporating all Phase 1 revision agenda items. A focused specialist panel of five validators (Table 3) was convened, comprising four neonatologists and one pediatric cardiologist drawn from five geographically distributed institutions across India and internationally. Validators were assigned sequential role codes (N1, PC1, N2, N3, E1); these codes denote order of participation and do not imply specialty classification.

**Table 3:** Phase 2 specialist validator panel. Role codes denote the sequential order of participation. “E1” designates the fifth enrolled specialist, not a specialty classification. Panel: four neonatologists + one pediatric cardiologist; five institutions across four Indian cities (Jaipur, Calicut, Chennai, New Delhi) and one international center (Muscat). Full names are provided in the Acknowledgements section. CVI = Content Validity Index

Code	Specialty	Name	Designation and institution	Role in validation
N1	Neonatology	Dr. Gunjana Kumar	Neonatologist, NIMS, Jaipur	Independent CVI reviewer: Protocol content
PC1	Pediatric Cardiology	Dr. Mirza Mohammad Kamran	Pediatric Cardiologist, Metromed, Calicut	Independent CVI reviewer: Cardiac accuracy
N2	Neonatology	Dr. Umamaheswari Balakrishnan	Professor & Head, Neonatology, Sri Ramachandra University, Chennai	Independent CVI reviewer: Protocol content
N3	Neonatology	Dr. Prakash Manikoth	Neonatologist, Muscat, Oman	Independent CVI reviewer: Protocol content
E1	Neonatology	Dr. Bharti Yadav	Assistant Professor, Neonatology, Safdarjung Hospital, New Delhi	Independent CVI reviewer: Protocol content

The Phase 2 instrument comprised 16 items mirroring the Likert domains of Phase 1, enabling direct comparison of I-CVI trajectories between phases. Modified kappa (k*) was calculated per item to adjust for chance agreement, using the formula described by Polit et al. [15]: k* = (I-CVI − 1/n)/(1 − 1/n), where n = number of validators. An item-level k* ≥0.74 was considered the minimum acceptable threshold for good agreement, with k* ≥0.80 indicating excellent agreement, per Polit et al. [15].

Phase 3: Author-led enhancement integration

Phase 3 involved no external validators. Following the unanimous Phase 2 endorsement, the principal innovator (GG) conducted a systematic evidence-referenced review to integrate eight clinical enhancements identified as substantive additions beyond the scope of the original Phase 1 revision agenda. Each enhancement was referenced against current international guidelines (Academy of Pediatrics (AAP), Neonatal Resuscitation Program (NRP), Pediatric Advanced Life Support (PALS), American Heart Association (AHA), European Society of Cardiology (ESC), 2023-2025). All changes were documented in a traceable revision log. The revised protocol was designated GiriMok V3.0.

Phase 4: Principal innovator quality assurance

Phase 4 constituted a final expert clinical review by the principal innovator (GG) to identify and correct any residual precision errors before publication submission. Five clinical precision corrections were identified and applied to produce GiriMok V4.0, the final publication-ready version.

AI assistance disclosure

Claude Sonnet 4.6 (Anthropic, PBC, San Francisco, CA) was used as an AI-assisted drafting tool for manuscript preparation, formatting support, structuring tables, CVI computation documentation, document generation, and title formulation [19]. All protocol development, expert validation processes, data verification, interpretation of findings, manuscript revision, and final scientific judgments were performed independently by the human authors. All clinical decisions, validation judgements, protocol content, and scientific interpretations are the sole responsibility of the principal author.

## Results

Phase 1: Broad multidisciplinary validation

Validator Panel Characteristics

In total, 18 external validators completed the Phase 1 assessment. The panel comprised nine neonatologists (50.0%), six pediatricians (33.3%), two pediatric cardiologists (11.1%), and one adult cardiologist (5.6%), representing a genuinely multidisciplinary composition. The mean clinical experience at the specialist level was 3.6 years (range = 3-6 years). All 18 validators declared no conflict of interest (100%).

Quantitative Content Validity Index Results

Item-level and S-CVI/Ave results for Phase 1 are presented in Table 4. S-CVI/Ave for the 16-item Likert matrix was 0.927, exceeding the pre-specified acceptance threshold of ≥0.90. All 16 items individually met the I-CVI ≥0.78 acceptability threshold. Two items achieved a perfect I-CVI of 1.000 (medication dosing accuracy and safety; color scheme appropriateness and accessibility). Seven items achieved an I-CVI of 0.944. Seven items yielded an I-CVI of 0.889, falling below the 0.90 sub-threshold, constituting the targeted revision agenda for V2.0 development.

**Table 4:** Phase 1 item-level CVI results (n = 18 validators). Threshold: I-CVI ≥ 0.78 per item; S-CVI/Ave ≥ 0.90. Sub-threshold items (I-CVI 0.889) identified the revision agenda for V2.0. n = 18 for quantitative items; n = 17 for categorical/qualitative items (one validator completed the Likert matrix only). I-CVI = item-level Content Validity Index; S-CVI/Ave = scale-level Content Validity Index; POC = point of care

Domain	Item	Ratings ≥ 3/18	I-CVI	Status
Content validity	Clinical accuracy of information	17/18	0.944	Optimal
	Current evidence alignment	16/18	0.889	Sub-threshold
	Diagnostic criteria completeness	17/18	0.944	Optimal
	Management protocol appropriateness	17/18	0.944	Optimal
	Medication dosing accuracy and safety	18/18	1.000	Perfect
	Completeness of coverage	16/18	0.889	Sub-threshold
Visual design	Visual clarity and readability	17/18	0.944	Optimal
	Information hierarchy and organisation	17/18	0.944	Optimal
	Colour scheme appropriateness	18/18	1.000	Perfect
	Graphics/Illustrations quality	16/18	0.889	Sub-threshold
Clinical usability	POC utility and practicality	17/18	0.944	Optimal
	Algorithm/flowchart clarity	16/18	0.889	Sub-threshold
	Ease of navigation	17/18	0.944	Optimal
Educational value	Teaching/Learning appropriateness	16/18	0.889	Sub-threshold
	Learning objective alignment	16/18	0.889	Sub-threshold
Overall assessment	Overall quality and publication readiness	16/18	0.889	Sub-threshold
	S-CVI/Ave		0.927	Accepted (≥0.90)

Evidence-Based Content Checklist Results (n = 17)

Currency of references: 82.4% affirmed. Evidence level documentation: 76.5% Yes. International guideline alignment: 88.2% Yes. Language appropriateness, cultural sensitivity, and freedom from jargon: unanimously affirmed (17/17). Zero safety concerns and zero factual errors were identified.

Safety Assessment

Zero validators identified any safety concern in GiriKous V1.0 (0/17), confirming the protocol’s foundational clinical safety before revision.

Clinical Application Recommendations (n = 17)

Across all four recommendation categories (clinical use, education, protocol development, publication), all 17 validators gave affirmative or conditional-affirmative responses; none withheld a recommendation in any category. Recommended for clinical use outright: 82.4%; for publication: 76.5%.

Qualitative Thematic Analysis

Qualitative analysis generated 22 revision items across four themes: Theme one (seven items) - factual corrections, including deemphasis of the hyperoxia test, updated AAP 2025 pulse oximetry criteria, and corrected “blue and comfortable = cardiac” teaching point; Theme two (seven items) - essential missing content, including POC echocardiography views, PGE₁ dosing table, arrhythmia management, and pre/post-ductal SpO₂ integration; Theme three (five items) - structural barriers to POC use, including the absent abbreviation glossary and lack of stepwise algorithmic pathways; Theme four (three items) - CTVS referral criteria, QR-linked references, and pericardiocentesis guidance. Strengths noted: pathophysiology-based structure, color-coded emergency prioritization, and multidisciplinary user-friendliness.

Phase 2: Focused specialist revalidation Content Validity Index results

Phase 2 yielded unanimous perfect content validity. All 16 items achieved an I-CVI of 1.000 and an S-CVI/Ave of 1.000. Modified kappa (k*) was 1.000 for all items, confirming that perfect agreement was not attributable to chance. The overall panel mean item rating was 3.66/4.00, reflecting strong agreement across all five validators. Zero corrective recommendations were generated. Phase 2 constituted a full endorsement of GiriMok V2.0 as submitted. Phase 1 versus Phase 2 CVI trajectories are presented in Table 5.

**Table 5:** Phase 1 versus Phase 2 CVI comparison: item trajectory. All seven previously sub-threshold items (I-CVI = 0.889 in Phase 1) achieved a perfect I-CVI = 1.000 in Phase 2, confirming complete resolution of the Phase 1 revision agenda. Phase two k* = 1.000 for all 16 items, exceeding the minimum acceptable threshold of k* ≥ 0.74 (Polit, Beck & Owen, 2007 formula). Overall panel mean rating = 3.66/4.00. Note: I-CVI computation treats all ratings ≥3 as relevant (binary threshold); the mean Likert rating of 3.66/4.00 reflects the distribution of actual scores within this threshold-satisfying range, confirming discriminative rather than acquiescent scoring. I-CVI = item-level Content Validity Index; S-CVI/Ave = scale-level Content Validity Index

Item	Phase 1 I-CVI	Phase 2 I-CVI (k*)	Δ I-CVI	Status
Clinical accuracy of information	0.944	1.000	+0.056	Improved to perfect
Current evidence alignment	0.889	1.000	+0.111	Improved to perfect
Diagnostic criteria completeness	0.944	1.000	+0.056	Improved to perfect
Management protocol appropriateness	0.944	1.000	+0.056	Improved to perfect
Medication dosing accuracy and safety	1.000	1.000	0.000	Maintained perfect
Completeness of coverage	0.889	1.000	+0.111	Improved to perfect
Visual clarity and readability	0.944	1.000	+0.056	Improved to perfect
Information hierarchy and organisation	0.944	1.000	+0.056	Improved to perfect
Colour scheme appropriateness	1.000	1.000	0.000	Maintained perfect
Graphics/Illustrations quality	0.889	1.000	+0.111	Improved to perfect
POC utility and practicality	0.944	1.000	+0.056	Improved to perfect
Algorithm/flowchart clarity	0.889	1.000	+0.111	Improved to perfect
Ease of navigation	0.944	1.000	+0.056	Improved to perfect
Teaching/Learning appropriateness	0.889	1.000	+0.111	Improved to perfect
Learning objective alignment	0.889	1.000	+0.111	Improved to perfect
Overall quality and publication readiness	0.889	1.000	+0.111	Improved to perfect
S-CVI/Ave	0.927	1.000	+0.073	Unanimous perfect

Phase 2 Qualitative Findings

All five specialist validators provided narrative feedback. Qualitative themes included high commendation for the systematic sequential structure; appreciation of the five C’s differential framework as a mnemonic aid; specific endorsement of the PPHN vs. cyanotic CHD differentiation section; suggestions for enhanced dosing tables for PGE₁ (incorporated in Phase 3); and recommendations for emphasis on echocardiographic limitations in resource-limited settings (incorporated in Phase 3). No validator raised safety concerns or identified factually incorrect clinical information.

Phase 3: Author-led enhancement integration

Eight evidence-referenced clinical enhancements were identified and integrated by the principal innovator (GG) to produce GiriMok V3.0. Enhancements were substantive additions beyond the Phase 1 revision scope, each referenced against current international guidelines (AAP, ESC, AHA, American Thoracic Society (ATS), American Thoracic Society (ATS), NRP, PALS, 2023-2025). These included expanded arrhythmia recognition and management; PPHN versus CCHD differentiation framework; resource-limited setting adaptations; CTVS referral criteria; postoperative monitoring guidance; long-term neurodevelopmental follow-up principles; pericardial effusion and pericardiocentesis reference; and QR-linked dynamic reference infrastructure. All enhancements were documented in a traceable revision log.

Phase 4: Principal innovator quality assurance

Five clinical precision corrections were identified and applied during the Phase 4 expert review to produce the final GiriMok V4.0 (Table 6).

**Table 6:** Phase 4 clinical precision corrections (principal innovator QA). All five corrections pertain to the GiriMok V4.0 clinical protocol tool. Following incorporation, a zero-outstanding corrections declaration was issued. V4.0 is designated as final and publication-ready. TAPVC = total anomalous pulmonary venous connection; TR = tricuspid regurgitation; POC = point of care; PGE₁ = prostaglandin E₁

Item corrected	Correction applied
Snowman sign: TAPVC	Corrected to: Snowman sign is absent in obstructed TAPVC; present only in unobstructed TAPVC. Prevents common and potentially dangerous radiological misinterpretation
Ebstein’s anomaly diagnostic criteria	Updated to body surface area-indexed echocardiographic criteria, ensuring diagnostic accuracy across neonates of varying gestational age
Great vessel relationship: echo nomenclature	Corrected echocardiographic view nomenclature for great vessel relationship assessment in the POC echo section
TR jet measurement units	Standardised tricuspid regurgitation jet measurement to correct units of measurement (m/s)
Pre/Post-ductal saturation table	Revised table entries for accuracy. PGE₁ absolute contraindication (obstructed TAPVC) confirmed as single entry; relative precautions delineated separately

Correction accounting across all phases

Total expert-informed corrections: 35 (Phase 1: 22 from external validators; Phase 2: zero, unanimous endorsement; Phase 3: eight author-led enhancements; Phase 4: five precision corrections = 35 total). Phase 2, generating zero new corrections, confirms that the Phase 1 revision agenda was comprehensively addressed and constitutes the expected outcome of a well-executed iterative validation process.

## Discussion

The GiriMok Protocol addresses a clinically important gap by providing a structured bedside approach for the evaluation and stabilization of cyanotic neonates. Beyond its validated content framework, the protocol has potential clinical utility as a rapid decision-support tool for frontline clinicians, particularly in resource-limited neonatal emergency settings where timely recognition and stabilization may improve early management and referral outcomes.

Principal findings and clinical significance

At 2 am in a Level II neonatal intensive care unit (NICU) without an echocardiographer, a frontline clinician facing a cyanotic neonate must make time-critical decisions: initiate PGE₁ or withhold it, transfer or stabilize, cardiac or pulmonary cause with limited subspecialty support, and, as the present study establishes, no known previously validated comprehensive bedside reference. The GiriMok Protocol was developed precisely for this clinical reality, and the present study establishes its content validity through a rigorous four-phase iterative methodology that produced a measurable, traceable improvement trajectory from S-CVI/Ave = 0.927 to S-CVI/Ave = k* = 1.000.

Three principal findings warrant discussion. First, strong initial content validity was established across a broad 18-member multidisciplinary panel, with unanimous safety endorsement. Second, following systematic incorporation of a 22-item expert-generated revision agenda, unanimous specialist endorsement was achieved at Phase 2 revalidation, with perfect CVI and k* values confirming agreement beyond chance. Third, the explicit reporting of all four phases, including author-led enhancement and quality assurance phases, provides a complete and transparent audit trail of tool development, constituting a methodological contribution that extends beyond the GiriMok Protocol itself.

Contextualization against existing validation literature

Content validity methodology, as operationalized through the CVI framework of Lynn [13] and Polit and Beck [14], has been applied across a range of neonatal and pediatric clinical tools in recent years, providing a verifiable benchmark. In the neonatal domain, Manzo et al. [20] reported CVI scores exceeding 0.90 across all items for a Safe Nursing Care Checklist for NICU infants, validated by 43 expert NICU nurses across three rounds [20]. Manzotti et al. [21] reported an S-CVI/Ave of 0.95 for the Neonatal Assessment Manual Score (NAME), a clinical management tool for NICU infants [21]. Silvino et al. [22] validated the FARNNeo Neonatal Nutritional Risk Screening Tool across three iterative Delphi cycles, achieving a CVI >0.90 for all items at the final cycle, notably requiring three revision cycles before the threshold was reached for one contested item [22]. Bibl et al. [23] validated the NeoCheck checklist for Newborn Life Support performance assessment using 12 international experts across three Delphi rounds, with all 38 final items achieving threshold [23]. Ruhl et al. [24] reported a two-round validation of the Maternal Fetal Triage Index (MFTI) by 45 multidisciplinary validators, achieving an S-CVI of 0.95 at Round 2.

Across these comparator studies, published S-CVI values for neonatal and perinatal clinical tools range from 0.87 to 0.95 at final or second-round validation. The Phase 1 S-CVI/Ave of 0.927 falls at the upper end of this verified range, confirming consistency with best-published neonatal tool validation data. A systematic search of PubMed and Scopus using terms including “cyanotic congenital heart disease,” “neonatal,” “point-of-care protocol,” and “content validation” identified no known published study reporting content validity evaluation of a comprehensive POC management protocol for the undifferentiated cyanotic neonate. The present study, therefore, addresses a validated gap that the existing evidence base confirms but has not previously filled.

Interpretation of Phase 1 Content Validity Index results

The Phase one S-CVI/Ave of 0.927 across 18 multidisciplinary validators on first submission is consistent with the upper range of published benchmark values for comparable neonatal clinical tools. Two items achieved a perfect I-CVI of 1.000, i.e., medication dosing accuracy and color scheme, confirming foundational unanimity on the two domains most directly implicated in patient safety and rapid visual recognition. The unanimous safety endorsement (0/17 validators identifying any safety concern) is a finding not universally reported as an explicit primary outcome in published tool validation studies, yet arguably the most clinically consequential finding a validation study of a clinical management tool can report.

The seven sub-threshold items (I-CVI = 0.889) spanned all five instrument domains, confirming that validators were calibrating completeness and usability rather than questioning clinical foundations, medication dosing accuracy and color scheme both achieved a perfect I-CVI of 1.000.

The four qualitative themes collectively confirm that the revision agenda was a calibration toward clinical excellence, not remediation of fundamental error: factual updates and missing content (Themes 1-2, 14 items) reflect the pace of change in neonatal cardiology; structural barriers to POC use (Theme 3) address the gap between clinical correctness and genuine bedside usability.

Interpretation of Phase 2 Perfect Content Validity Index: Reframing the narrative

The achievement of S-CVI/Ave = k* = 1.000 at Phase 2 is best understood not as a statistical anomaly requiring explanation but as the methodologically coherent anticipated outcome of a well-designed iterative validation process. Phase 2 assessed GiriMok V2.0, a protocol that had comprehensively incorporated all 22 Phase 1 revision agenda items using a focused specialist panel with direct clinical expertise in the tool’s target domain. When a rigorous revision process addresses expert-identified deficiencies and the revised tool is then assessed by the most clinically relevant validators.

The two-tier panel design reflects a deliberate methodological strategy. Phase 1’s broad panel of 18 validators captured the full range of clinical perspectives relevant to the target user population, including frontline pediatricians, the largest group of first contact clinicians for cyanotic neonates in resource-limited settings. Phase 2’s focused panel of five specialists achieved domain-specific consensus from the validators with the greatest clinical authority to endorse the revised protocol. This sequential architecture is consistent with recommended approaches for iterative content validation in specialist clinical domains and is directly analogous to the two-round design of Ruhl et al. [24].

The k* of 1.000 confirms agreement was not attributable to chance, methodologically essential given I-CVI is constrained to discrete 0.20 steps with five validators. The mean item rating of 3.66/4.00 confirms discriminative scoring rather than ceiling-effect acquiescence.

Methodological contribution: Transparent four-phase iterative reporting

The four-phase architecture of the present study contributes a replicable methodological framework to the clinical tool validation literature. Most published CVI-based validation studies report a single round of expert review, with revision processes described summarily rather than phase explicitly. The present study explicitly distinguishes and separately reports all four phases, enumerating all 35 expert-informed corrections with phase-specific provenance. This level of transparency is consistent with reproducibility standards recommended by the COSMIN framework for clinical measurement instrument development [25], and provides an audit trail that would support independent replication.

Clinical applicability, generalizability, and health equity implications

The multidisciplinary Phase 1 panel composition ensures content validity extends across the full range of clinicians who encounter cyanotic neonates in first-contact settings, not merely subspecialists. The geographical diversity of the Phase 2 panel, with five institutions across four Indian cities and one international center, supports generalizability beyond a single institutional context.

No existing validated tool integrates all five functions essential at first contact for the undifferentiated cyanotic neonate bedside screening, differential diagnosis, emergency pharmacotherapy, diagnostic interpretation, and transfer decision-making, a structural gap confirmed by the present literature search.

CHD approximately 8-12 per 1,000 live births in India, with cyanotic lesions accounting for approximately 20% of cases [2,9], and the majority of affected neonates present to facilities without on-site pediatric cardiology, the precise deployment context for which the GiriMok Protocol is designed. From a health equity perspective, a validated, freely disseminable POC tool that enables frontline clinicians in resource-limited settings to manage cyanotic neonatal emergencies with the same systematic rigor available in tertiary centers represents a meaningful step toward reducing the well-documented survival gap for CHD in LMICs.

Despite strong content validity, implementation of the GiriMok Protocol in real-world neonatal practice may face challenges related to variability in clinician expertise, infrastructure limitations, and resource availability, particularly in LMIC and non-tertiary settings. Accurate application of components such as pre/post-ductal SpO₂ interpretation, hyperoxia testing, PGE₁ administration, and focused POC echocardiography requires foundational neonatal critical care training and competency-based supervision. Additional barriers may include inconsistent access to infusion pumps, echocardiography equipment, arterial blood gas analysis, neonatal transport systems, and uninterrupted PGE₁ availability. The protocol is intended to support rather than replace clinical judgement, and subspecialty consultation remains essential for definitive diagnosis and management. Safe implementation will, therefore, require structured educational strategies including simulation-based training, case-based learning, supervised bedside mentoring, and integration into neonatal resuscitation and continuing medical education programs. Formal dissemination pathways, including NRP instructor networks and open-access digital platforms, should be explored upon prospective clinical validation.

Limitations

The four-phase iterative design, multidisciplinary panel composition, transparent correction accounting, and zero safety concern finding represent substantive methodological strengths. The following limitations should nonetheless be considered.

Content Validity Versus Clinical Effectiveness

Content validity, however rigorously established, does not establish clinical effectiveness. The GiriMok Protocol’s impact on patient outcomes, time to diagnosis, PGE₁ initiation accuracy, transfer appropriateness, and survival requires prospective evaluation. This is the primary limitation and defines the most urgent research priority.

Single-Institution Protocol Development

While external validation involved geographically diverse validators, the protocol architecture was developed by a single innovator at a single institution. Multicenter co-development in future iterations would further strengthen generalizability.

Validator Seniority

The mean specialist experience of Phase 1 validators was 3.6 years. While early-career specialists may better reflect the target frontline user population, making this a representational asset as much as a limitation, a panel incorporating more senior validators would enrich the revision agenda from a different vantage point.

Phase 2 Panel Overlap and Independence

All five Phase 2 validators were drawn from the Phase 1 panel. While this design enables iterative re-assessment of responsiveness to feedback by the same expert cohort, it limits the independence of Phase 2 revalidation. Future studies should include independent specialist validators not involved in Phase 1 to fully separate the validation and endorsement functions.

Panel Size and I-CVI Mathematical Constraints

The Phase 2 panel of five validators constrains I-CVI to discrete values with no intermediate step between 0.80 and 1.00. A larger panel of 7-10 specialists would provide finer resolution and is recommended for multicentre revalidation.

Inter-rater Reliability and Volunteer Bias

No overall inter-rater reliability statistic was computed across the full rating matrix, which should be addressed in future studies. Volunteer bias is partially mitigated by the substantive corrective feedback documented across all qualitative categories, indicating genuine critical engagement.

## Conclusions

The GiriMok Protocol bridges a critical gap in newborn care. It gives frontline clinicians a validated, practical bedside reference that ties together five essential actions needed the moment a cyanotic newborn arrives: screening at the bedside, mapping out a differential diagnosis, starting emergency medications, interpreting diagnostic tests, and deciding when to transfer. By keeping these five core clinical functions in one clear framework, it ensures safer, faster management when every second counts.

## Appendices

The link to the protocol: https://drive.google.com/file/d/1Z7M276W7T_38X2rSI4sKDZis5wYTZOYL/view?usp=sharing.
